# Day-to-day physical activity producing low gravitational impacts is associated with faster visual processing speed at age 69: cross-sectional study

**DOI:** 10.1186/s11556-019-0216-3

**Published:** 2019-06-25

**Authors:** Ahmed Elhakeem, Kimberly Hannam, Kevin C. Deere, Andrew Wong, Tim Gaysin, Diana Kuh, Rachel Cooper, Marcus Richards, Jon H. Tobias

**Affiliations:** 1 Musculoskeletal Research Unit, Translational Health Sciences, Bristol Medical School, University of Bristol, Learning & Research Building Level 1, Southmead Hospital, Bristol, BS10 5NB UK; 2 MRC Integrative Epidemiology Unit at University of Bristol, Population Health Sciences, Bristol Medical School, Bristol, UK; 3 MRC Unit for Lifelong Health and Ageing at UCL, WC1B 5JU, London, UK

**Keywords:** Accelerometer, Birth cohorts, Cognition, Physical activity, Vertical impacts

## Abstract

**Background:**

Little is known about how different physical activity (PA) parameters relate to cognitive function in older adults. Using accelerometers calibrated to detect vertical impacts from ground reaction forces we examined the associations of low, medium and higher impact PA with processing speed, verbal memory and cognitive state in older adults.

**Methods:**

Participants were 69-year old British men and women from the Medical Research Council National Survey of Health and Development included in a vertical impacts and bone sub-study (*n* = 558; 48.2% female). Counts of low (0.5 < *g* < 1.0 *g*), medium (1 < *g* < 1.5 *g*), or higher (≥1.5 *g*) magnitude impacts were derived from vertical acceleration peaks recorded over 7 days by hip-worn accelerometers. Processing speed was assessed by a timed visual letter search task, verbal memory by a 15-word list learning test and cognitive state by the Addenbrooke’s Cognitive Examination (ACE-III). Potential confounders were childhood cognitive ability, adult socioeconomic position, body mass index and depression.

**Results:**

In initial sex-adjusted models, low magnitude impacts were associated with better performance in all three cognitive function tests; standard deviation differences in test scores per doubling in number of low impacts: letter search speed = 0.10 (95% confidence intervals (CI): 0.03 to 0.16), word learning test = 0.05 (95% CI: 0.00 to 0.11), ACE-III scale = 0.09 (95% CI: 0.03 to 0.14). After adjustment for confounders, differences persisted for letter search speed (0.09; 95% CI: 0.02 to 0.16) but were closer to the null for the word learning test (0.02; 95% CI: − 0.04 to 0.07) and ACE-III scores (0.04; 95% CI: − 0.01 to 0.09). Low impacts remained associated with letter search speed after sensitivity analyses excluding those with functional and musculoskeletal problems, and after adjustment for impacts in higher bands. Modest positive associations between higher magnitude impacts and cognitive test scores were most likely due to chance.

**Conclusion:**

Accelerometer-derived low impact physical activity was associated with better visual processing speed in 69-year old men and women independently of childhood cognitive ability and other measured confounders. Day-to-day low impact physical activity may therefore have the potential to benefit cognitive health in older adults.

**Electronic supplementary material:**

The online version of this article (10.1186/s11556-019-0216-3) contains supplementary material, which is available to authorized users.

## Background

Evidence suggests that physical activity (PA) improves brain health and can help reduce age-related cognitive decline [1–3]. The influences of PA on cognition are thought to operate via a number of different underlying pathways depending on specific parameter(s) of PA such as type and intensity. Aerobic exercise for instance is thought to be important for cognition [1–3] and studies using accelerometers to measure PA in older adults suggest that both light [4] and higher intensity PA [5] is associated with better cognitive function. Furthermore, different combinations of aerobic, force and coordination training may exert greater benefits than single exercises since the diverse aspects of such training have been shown to induce different brain and behavioural responses [1, 2]. Despite this, very little is known about the characteristics of PA that are most important for cognition and there is no consensus on the type of PA that is most beneficial [2, 6]. Moreover, when examining the influence of PA on cognition in later life, controlling for reverse causality by taking account of prior cognitive ability is important [7]; however, very few studies have these measures.

Whereas accelerometer outputs are conventionally classified according to intensity using thresholds calibrated against oxygen consumption, many other characteristics of PA can be evaluated using alternative approaches which can provide novel insights into its relationships to health outcomes. For example, a recent study using a novel and validated method for parameterising older adults’ accelerometer-measured PA according to level of vertical impact [8, 9] found positive associations between higher, but not lower, impact PA and bone strength in older women [10], supporting the hypothesis that PA needs to exceed a certain impact magnitude to benefit bone [11]. In contrast, only low impact PA was inversely related to body mass index (BMI) and fat mass in a multicohort study of older adults, likely reflecting effects of total activity on adiposity in this population [12].

To provide a deeper understanding of the relationship between PA and cognition, in the present study, we aimed to examine the associations between accelerometer-measured PA, classified according to level of vertical impacts, and cognitive performance in a population-based sample of older adults where prior cognitive ability was also assessed prospectively in childhood. We hypothesised that all PA regardless of impact magnitude (i.e. lower, medium and higher impact PA) would be associated with better cognitive performance and that these associations would be independent of early life cognitive ability.

## Methods

Study participants were from the Medical Research Council (MRC) National Survey of Health and Development (NSHD), a national sample initially consisting of 5362 British births occurring during 1 week in March 1946 that has to date been regularly followed-up to age 69 years [13]. Most participants (79%) included in the home visit phase of the NSHD 24th data collection in 2015–16 [13] were invited to participate in the Vertical Impacts on Bone in the Elderly (VIBE) study [8, 14], which was initially set up to investigate the health consequences of higher impact PA in older people. Relevant ethics approval has been granted for each data collection; ethical approval for the most recent assessment in 2014–2015 was obtained from the Queen Square Research Ethics Committee (14/LO/1073) and the Scotland A Research Ethics Committee (14/SS/1009). Study participants provided written informed consent.

During the home visit at age 69, participants were invited to participate in the VIBE study. If they agreed, the nurse provided them with a GCDC X15-1c triaxial accelerometer (Gulf Coast Data Concepts, Waveland, Mississippi), custom designed elasticated belt, a time log and a stamped addressed package along with instructions. Accelerometers were configured to a sampling frequency of 50 Hz, a deadband setting of 0.1 g and a timeout setting of 10 s. We instructed participants to wear the accelerometer securely positioned in the belt over their right hip pointing toward the centre of their body for seven continuous days, removing only for sleeping, washing and swimming. Participants were asked to record the times at which the monitor was put on in the morning and taken off at night for each monitoring day and to state reasons, if any, why that day had not been reflective of their normal activity.

Standardised cleaning and processing of raw accelerometer data was carried out by the study coordinating centre and is described in detail elsewhere [8]. Briefly, data were cleaned to remove movement artefacts and non-wear time, and activity data were normalised based on seven valid days of ≥10 h recording time. Vertical (i.e. Y-axis) accelerations peaks were then calculated based on accelerations higher than the preceding and subsequent reading. Participants were grouped into three bands reflecting low (0.5 < g < 1.0), medium (1.0 < g < 1.5 g) and higher (≥1.5 g) impact. The ≥1.5 g cut-point for higher impacts was selected as very few impacts were observed within higher g bands [8, 14]. Periods of inactivity were removed by excluding accelerations ≤0.5 g^8^. All g values represent above 1 g from earth’s gravitational force.

Cognitive function was assessed at age 69 by tests of processing speed and verbal memory, and by the Addenbrooke’s Cognitive examination-III (ACE-III) scale. Processing speed was assessed by a timed visual search task requiring cancellation of target letters P and W embedded among non-target letters; the speed score was derived from the position reached at the end of 1 minute. Verbal memory was assessed by a 15-word list learning task with three learning trials and free written recall at the end of each trial, therefore the maximum score achievable was 45. The ACE-III scale is the most comprehensive test of cognitive state, developed for use in clinical settings. It includes five subdomains that assess attention, memory, fluency, language and visuospatial ability, and has a maximum score of 100, with a quasi-normal distribution. Recent studies demonstrate the validity of ACE-III for diagnosing mild cognitive impairment, Alzheimer’s disease and dementia [15]. Each cognitive measure was standardised to mean = 0 and standard deviation (SD) = 1.

Childhood cognition, own socioeconomic position (SEP), and contemporaneous BMI and depression were identified as potential confounders. Childhood cognitive ability was tested at age 15 using the Heim AH4 test of verbal and non-verbal ability [16] Watts-Vernon reading comprehension test [17] and a test of mathematical ability [18]. Test scores were combined to derive an overall standardised score (mean = 0 and standard deviation (SD) = 1). Own SEP was based on highest Registrar General’s occupational class at age 53 years (and if missing, then taken from earlier ages), categorised as professional or intermediate; skilled non-manual; skilled manual; and semi-skilled or unskilled manual. BMI (kg/m^2^) was calculated from heights and weight measured by nurses at age 69; heights were measured to the nearest millimetre using a Leicester stadiometer (Marsden Group, UK) and weights to the nearest 100 g using Tanita weighing scales (Tanita UK Ltd., Uxbridge, UK).

Depression was assessed at age 69 using responses to questions in the depression subscale of the General Health Questionnaire-28, a screening tool used to detect risk of psychiatric disorders [19]. Responses to each question (Been thinking of yourself as a worthless person? Felt that life is entirely hopeless? Felt that life isn’t worth living? Though of the possibility that you might make away with yourself? Found at times you couldn’t do anything because your nerves were too bad? Found yourself wishing you were dead and away from it all? Found that the idea of taking your own life kept coming into your mind?) were assigned a score (0 = not at all, 1 = no more than usual, 2 = rather more than usual, 3 = much more than usual) and summed to derive a total score with potential range from 0 to 21.

We initially examined how vertical impacts related to cognitive function by plotting mean scores for each cognitive test across quartiles of low, medium and high impacts, and tested trends using an extension of the Wilcoxon rank-sum test (Cuzick’s test for trend). Separate linear regression models were then used to examine associations between each PA impact measure (low, medium and high impacts) and each cognitive score. Accelerometer data were log-transformed due to their skewed distributions, and model estimates presented as SD difference in each cognitive score per doubling in the number of impacts. Interaction terms were used to test sex differences, and subsequently men and women were combined, with adjustment made for sex after little evidence of interaction was found. Three models were fitted to test associations between PA within each impact band and each cognitive score; a sex-adjusted model which was subsequently further adjusted for SEP, BMI and depression, and then additionally for childhood cognition. Models were fitted after multiple imputation of missing confounders (*n* = 72 participants) using 20 multiply imputed datasets which were combined with Rubin’s combination rules [20]. Analyses were performed in STATA 15.

We investigated if any associations found for specific impact levels were independent of total PA by fitting additional models with mutual adjustment for PA within other impact bands. We also examined if musculoskeletal or functional problems influenced findings by repeating the main analyses after excluding in turn those with difficulties walking i.e. noticeable limp (*n* = 57), walking restricted due to pain (*n* = 114), regular mobility aid use (*n* = 29), falls in the past year (*n* = 136) and fractures since age 45 (*n* = 231). This information was captured by a self-reported questionnaire left with participants to complete and return with their accelerometer. Finally, we compared multiple imputation results to complete-case analyses.

## Results

**Fig. 1:**
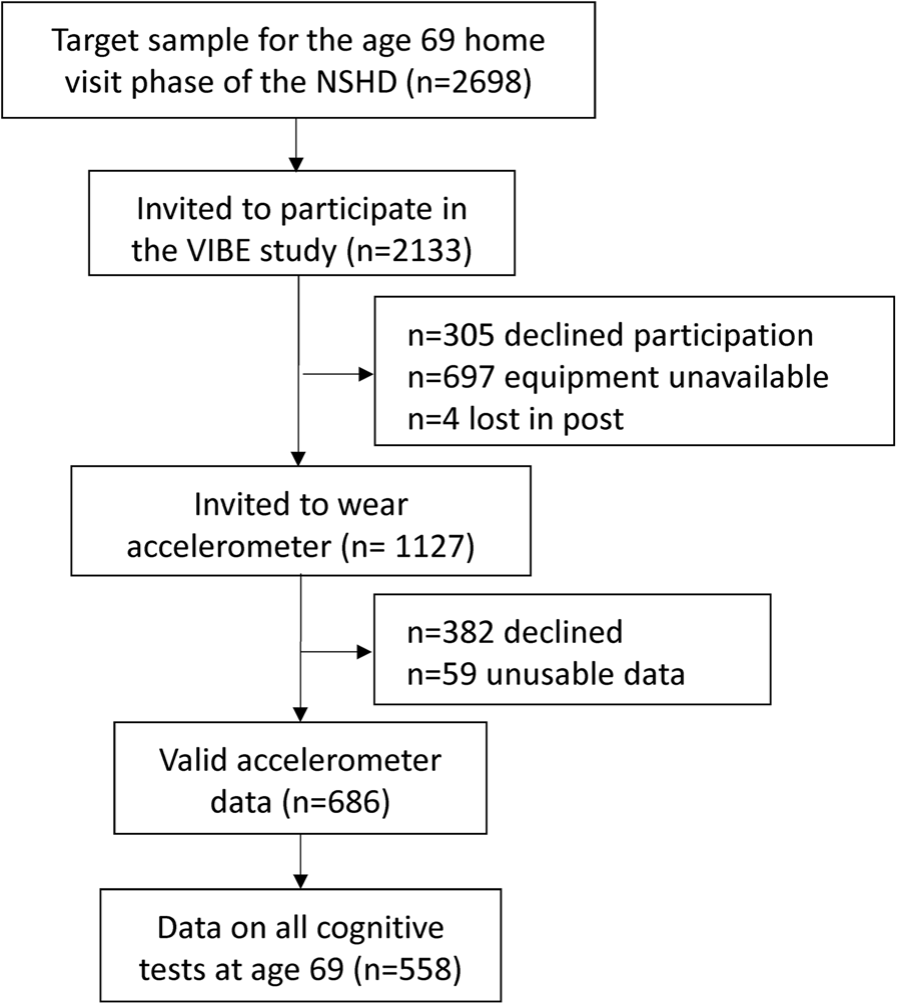
Study flowchart

Of the 1127 invited to wear an accelerometer, 686 had valid PA data, and of these, 558 (48.2% female) had data on adult cognitive scores. (Table 1, Fig. 1). When compared to those without accelerometer data, those with valid accelerometer data had better childhood cognition (mean z-score: 0.29 vs. 0.11) and lower adult BMI (27.3 vs. 28.6 kg/m^2^) and depression scores (0.67 vs. 0.84) and slightly better adult cognitive scores (letter search task: 263.6 vs. 261.7; word learning test: 23.0 vs.21.8; ACE-III: 92.4 vs. 91.1). Higher proportions of those with valid accelerometer data were in the most advantaged occupational classes (53.5% vs. 45.1%). Vertical impacts from PA comprised 94.3% low magnitude impacts (0.5 < g < 1.0), 5.2% medium impacts (1.0 < g < 1.5 g), and only 0.5% higher impacts (> 1.5 g); men accumulated higher counts of low, medium and higher impacts than women (Table 1).

**Table 1:** Characteristics of participants from the MRC National Survey of Health and Development with data from accelerometry and cognitive tests at age 69, 2015 (*n* = 558)

	Men (*n* = 289)	Women (*n* = 269)
*Cognitive test scores: mean (SD)*		
Letter search task	253.2 (72.5)	274.2 (75.0)
Word learning test	22.0 (5.8)	24.1 (6.0)
Addenbrooke’s Cognitive examination-III	92.1 (5.2)	92.6 (5.4)
*Vertical acceleration peaks: Median count (25th, 75th)*		
Low magnitude impacts (0.5 ≤ g < 1.0)	15,909 (7800, 25,888)	13,513 (7228, 23,301)
Medium magnitude impacts (1.0 ≤ g < 1.5)	968 (305, 2691)	667 (243, 1753)
Higher magnitude impacts (≥ 1.5 g)	101 (42, 315)	70 (29, 192)
*Adult socioeconomic position: N (%)*		
professional and intermediate	170 (59.2)	131 (48.7)
skilled non-manual	30 (10.5)	94 (34.9)
skilled manual	69 (24.0)	13 (4.8)
semi-skilled and unskilled manual	18 (6.3)	31 (11.5)
BMI (kg/m^2^): mean (SD)	27.5 (3.9)	27.2 (4.5)
Depression score from the GHQ-28 score: mean (SD)	0.58 (1.8)	0.79 (1.8)
Childhood cognitive ability: mean (SD)	0.06 (1.02)	−0.06 (0.98)

*SD* standard deviation, *PA* physical activity, *BMI* body mass index, *GHQ* general health questionnaire. Childhood cognitive ability score standardised to mean = 0 and SD = 1

**Fig. 2:**
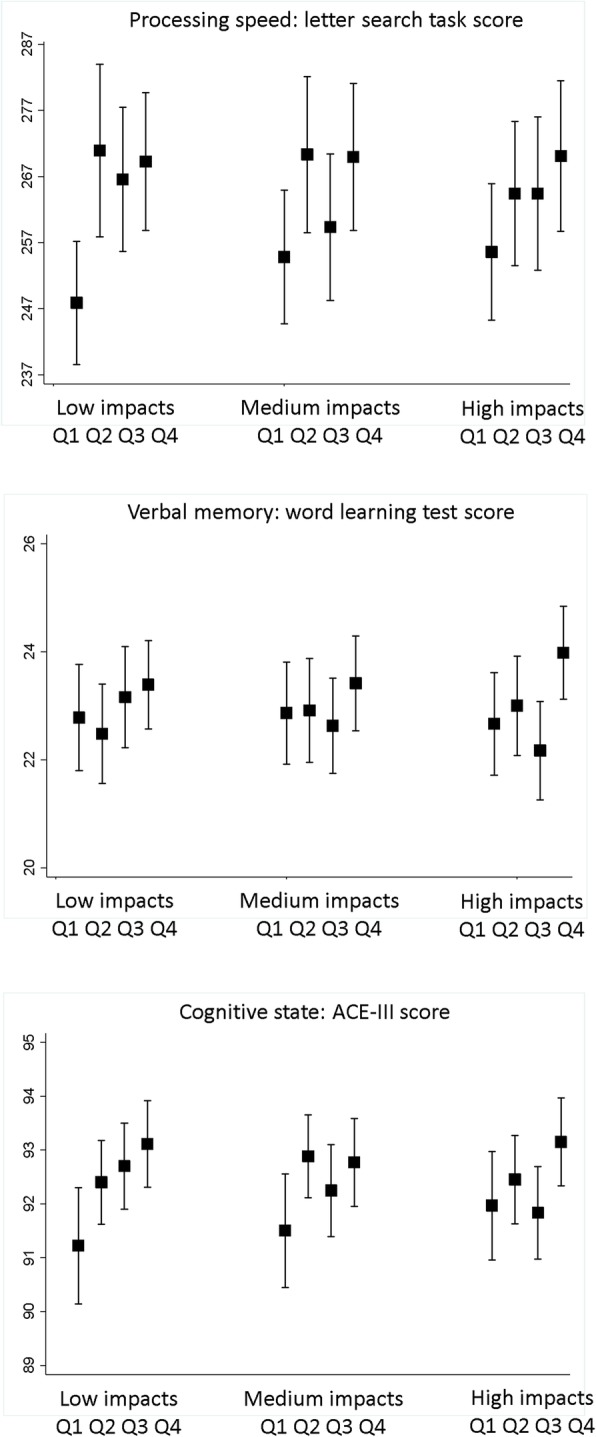
Mean cognitive test scores by quartile of low (0.5 ≤ g < 1.0), medium (1.0 ≤ g < 1.5) and higher (≥ 1.5 g) impact counts. Vertical bars reflect 95% confidence intervals

There were trends of better performance in the letter search task and the ACE-III across higher quarters of impacts; however, differences were greater for low impacts than medium or higher impacts (Fig. 2). Difference in mean word letter search scores between highest and lowest quarters were 21.4 for low impacts (*P* trend =0.01), 15.1 for medium impacts (*P* trend = 0.2) and 14.5 for higher impacts (*P* trend = 0.2*).* The equivalent differences in mean ACE-III scores were 1.9 for low impacts (*P* trend = 0.02) and 1.2 for both medium and high impacts (*P* trend = 0.3). Similar but less pronounced trends were observed for word learning test scores; equivalent differences were 0.61 for low impacts (*P* trend = 0.1), 0.55 for medium impacts (*P* trend = 0.5) and 1.3 for high impacts (*P* trend = 0.5).

**Fig. 3:**
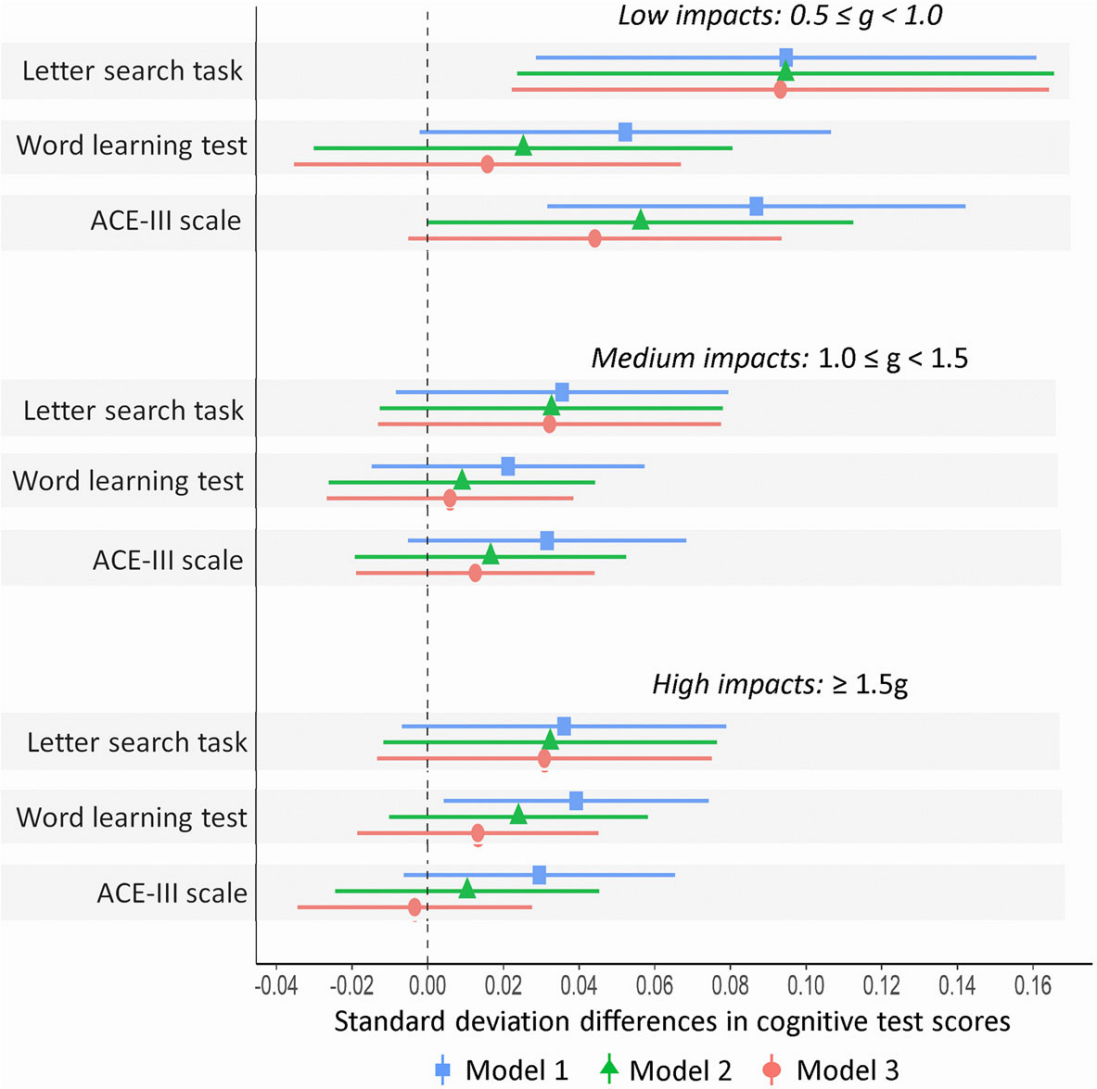
Standard deviation difference in cognitive test scores per doubling in number of low (0.5 ≤ g < 1.0), medium (1.0 ≤ g < 1.5) and higher (≥ 1.5 g) magnitude impacts (*n* = 558). Model 1: adjusted for sex. Model 2: adjusted for sex, socioeconomic position, body mass index and depression. Model 3: as for model 2 plus adjustment for early life cognition. Horizontal bars reflect 95% confidence intervals

Figure 3 represents the multivariable models. In Model 1 (sex-adjusted), greater counts of low impacts (0.5 < g < 1.0) were associated with better performance on all three cognitive tests (SD differences were: letter search task = 0.10 (0.03 to 0.16), word learning test = 0.05 (0.00 to 0.11), ACE-III = 0.09 (0.03 to 0.14). Adjustment for SEP, BMI and depression (Model 2) had little effect on the association with letter search task (SD differences per increasing number of low impacts = 0.10; 0.02 to 0.18) and considerably attenuated the associations with the ACE-III scale (0.06; 0.00 to 0.11) and the word learning test (0.03; − 0.03 to 0.08). Further adjustment for early life cognitive ability (Model 3) only slightly attenuated these associations (letter search speed = 0.09; 0.02 to 0.16, word learning test = 0.02; − 0.04 to 0.07, ACE-III = 0.04; − 0.01 to 0.09).

Greater counts of high impacts (> 1.5 g) were associated with modestly higher scores on the word learning test (0.04; 0.00 to 0.07), letter search task (0.04; − 0.01 to 0.08) and the ACE-III scale (0.03; − 0.01 to 0.07) in sex-adjusted analyses (Fig. 3). These association were slightly attenuated after further adjustment in Model 2 for SEP, BMI and depression (word learning test = 0.02; − 0.01 to 0.06, letter search task = 0.03; 0.01 to 0.08, ACE-III = 0.01; − 0.02 to 0.05) and for childhood cognitive ability in Model 3 (word learning test = 0.01; − 0.02 to 0.05, letter search task = 0.03; − 0.01 to 0.08, ACE-III = 0.00; − 0.03 to 0.03). Medium impacts were not significantly associated with any cognitive outcome including in any model (Fig. 3).

The estimates of association between low impacts and cognitive scores strengthened but confidence intervals were wider after mutual adjustment for medium and higher impact PA: letter search task = 0.18 (0.05 to 0.31), word learning test = 0.04 (− 0.06 to 0.13), ACE-III = 0.08 (− 0.01 to 0.17). The weak association between vertical impacts > 1.5 *g* and word learning test scores strengthened but remained weak after adjustment for low impacts (0.03; − 0.03-0.10).

Excluding those with functional/musculoskeletal problems generally had little influence on the findings. For example, fully-adjusted (i.e. for sex, SEP, BMI, depression and childhood cognition) SD difference in letter search task per doubling in number of low impacts were: no mobility aid use = 0.13 (0.02 to 0.24, *n* = 529), no noticeable limp = 0.10 (− 0.01 to 0.21, *n* = 501), walking unrestricted by pain = 0.11 (− 0.01 to 0.24, *n* = 444), no falls in the past year = 0.06 (− 0.07 to 0.18, *n* = 422), no fracture since age 45 = 0.12 (− 0.01 to 0.25, *n* = 327), The equivalent differences in letter search task score for high impacts were:, no mobility aid use = 0.05 (− 0.02 to 0.11, n = 529), no noticeable limp = 0.03 (− 0.04 to 0.10, *n* = 501), walking unrestricted by pain = 0.05 (− 0.03 to 0.12, *n* = 444), no falls in the past year = 0.04 (− 0.04 to 0.11, *n* = 422), no fracture since age 45 = 0.03 (− 0.05 to 0.11, *n* = 327). Finally, findings were broadly similar for participants with non-missing data on confounders (*n* = 486); though the association between higher impacts (> 1.5 g) and word learning test score was stronger in the reduced sample (Additional file 1).

## Discussion

This study used a novel accelerometer method to quantify exposure to PA in later life according to vertical impacts from ground reaction forces and examine their relation to contemporaneous cognition in 69-year old men and women from a British birth cohort. Our findings showed that PA producing low magnitude vertical impacts was positively related to cognitive performance, particularly faster processing speed and higher scores for cognitive state (ACE-III). These associations were independent of measured confounders including childhood cognition and were robust to exclusion of those with musculoskeletal and functional problems and to adjustment for higher impact PA. We also found evidence of positive but weak associations between impacts > 1.5 *g* and verbal memory.

This is the first study to objectively quantify physical activity according to level of vertical impacts from ground reaction forces and examine how both low and higher impact activity relates to cognitive performance in later life. Our finding that low impact PA was positively related to cognition is consistent with findings from a large study of Chinese older adults showing that regular low impact mind-body exercises like Tai Chi and yoga were associated with lower risk of dementia [21]. The weaker associations for medium and higher impacts are inconsistent with results showing that higher intensity PA is more strongly related to better cognition [5], where objectively measured PA was classified using intensity cut-points based on energy expenditure. However, low impact PA based on vertical impact magnitude as in our study may not necessarily be comparable with light intensity PA based on energy expenditure; for example, cycling is low impact but can be high intensity. Further, light intensity PA, which makes up the majority of time spent in PA among older adults [22], has been shown to be associated with improved cognition, including independently of higher intensity PA [4].

Nearly all PA in this study was low impact. We suspect that much of the PA producing low impacts in this population to be from lower intensity activities, predominantly walking. In that case, our results would be consistent with findings in older women showing that reported walking was associated with better cognitive performance [23] and that pedometer-assessed walking was related to larger hippocampal volume [24]. Moreover, multicomponent low impact exercises that involve explicitly challenging balance and coordination movements have been suggested as important for preserving cognitive function in older adults [1, 2, 25]. Despite similar direction of association, that results were stronger for low impacts and weaker for medium and higher impacts may be because lower impacts are better markers of overall PA in older adults or due to the limited prevalence as well as limited variability in medium and higher impacts [9, 10, 12].

Some of the mechanisms underlying the associations found between low impact PA and cognition may operate through direct influences of total PA on brain function as well as by preventing diseases that impair cognition [2]. These may include hippocampal and frontal cortex neurogenesis that is possibly mediated by molecular changes [6], and cerebrovascular involvement in cortical-subcortical circuitry linking PA and cognition [6] that is facilitated by PA effects on reduced cardiovascular risk [25] and subsequent prevention of cerebrovascular disease. Supporting the latter point is that low impact exercises like walking [26] and cycling [27] are recognised as being important for cardiovascular health.

A major strength of this study is the novel use of raw accelerometer data to parameterise PA according to vertical impact and provide new insights into associations with cognitive function in later life [28, 29]. Use of a cross sectional study design makes it impossible to exclude reverse causality; however, our main findings were maintained after accounting for early life cognition which supports the direction of association from low impacts to better cognition. This is further supported by evidence from animal studies of neurogenesis in rats following treadmill exercise [30]. Whilst we examined processing speed, verbal memory and overall cognitive state, we did not examine other components of executive function such as list organisation, dual-task management, and aspects of problem solving. VIBE participants tended to be healthier (e.g. of lower BMI and better health status) when compared with those who did not participate in VIBE [14], and those without accelerometer data differed from included participants in cognitive scores and model covariates; therefore selection bias is likely and could explain our findings. Unmeasured or imprecisely measured confounders might also influence our findings.

## Conclusions

In conclusion, we used raw accelerometer data to describe PA according to level of vertical impact and investigated its association with cognitive function in a 69-year old British birth cohort. We found that PA producing low magnitude impacts, which made up the majority of all PA in this older population, was associated with faster visual processing speed independently of childhood cognitive ability and other confounders. Further studies are required to determine the causal nature of this association.

## Additional file

Additional file 1: Standard deviation difference in cognitive test scores per doubling in number of low (0.5 ≤ g < 1.0), medium (1.0 ≤ g < 1.5) and higher (≥ 1.5 g) magnitude impacts in the reduced sample i.e. non-missing data on confounders (*n* = 486). Model 1: adjusted for sex. Model 2: adjusted for sex, SEP, BMI and depression. Model 3: as for model 2 plus adjustment for childhood cognition. Horizontal bars reflect 95% confidence intervals (CI). (TIF 1536 kb)

## Data Availability

Data used in this publication are available to bona fide researchers upon request to the NSHD Data Sharing Committee via a standard application procedure. Further details can be found at http://www.nshd.mrc.ac.uk/data. doi: 10.5522/NSHD/Q103.
