# Supplementary figures and images for: Day-to-day physical activity producing low gravitational impacts is associated with faster visual processing speed at age 69: cross-sectional study
Source: Eur Rev Aging Phys Act. 2019 Jun 25;16:9. doi: 10.1186/s11556-019-0216-3 (PMC6593499; doi:10.1186/s11556-019-0216-3)

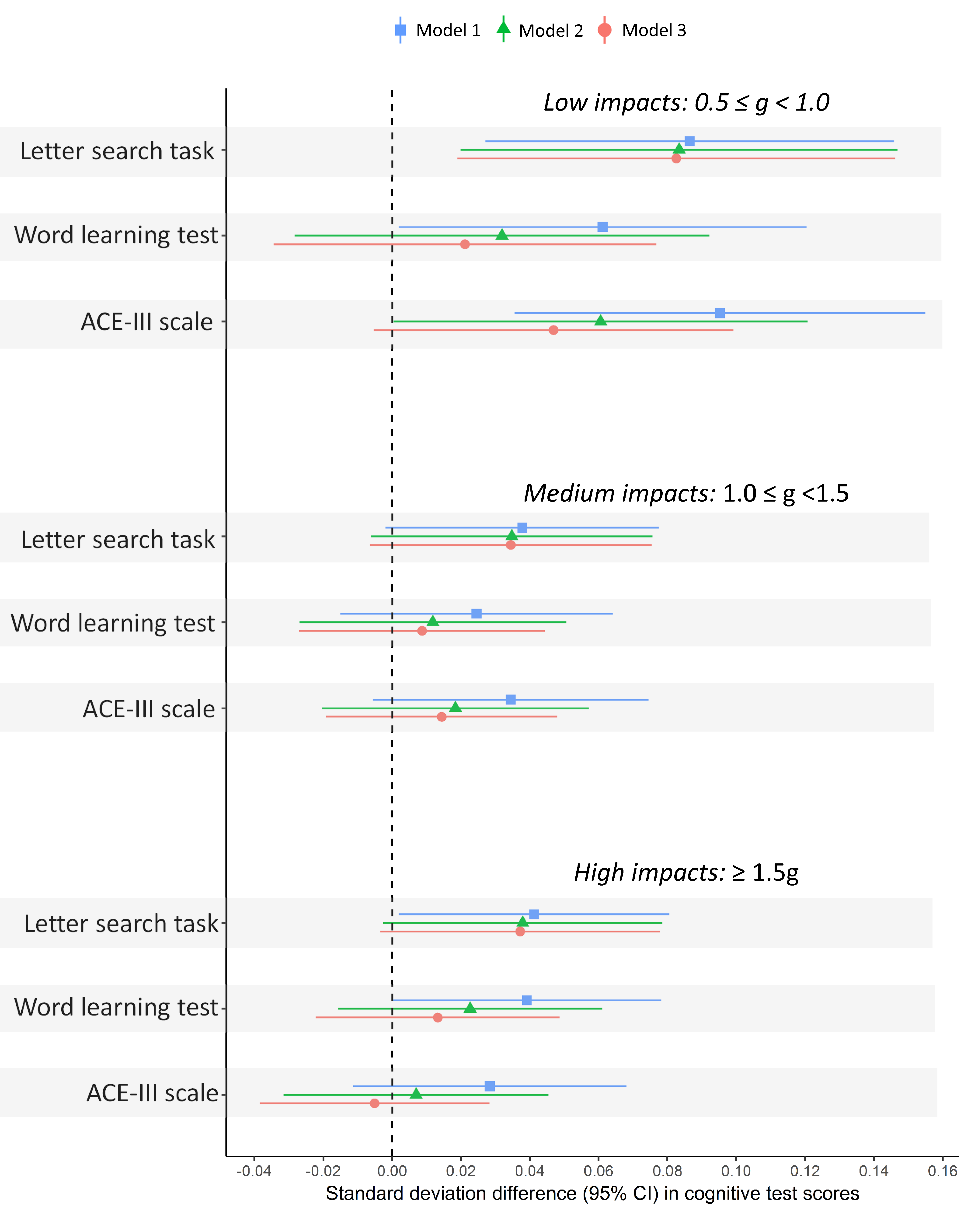

Supplement: Supplementary file 1 — Standard deviation difference in cognitive test scores per doubling in number of low (0.5 ≤ g < 1.0), medium (1.0 ≤ g < 1.5) and higher (≥ 1.5 g) magnitude impacts in the reduced sample i.e. non-missing data on confounders (n = 486). Model 1: adjusted for sex. Model 2: adjusted for sex, SEP, BMI and depression. Model 3: as for model 2 plus adjustment for childhood cognition. Horizontal bars reflect 95% confidence intervals (CI). (TIF 1536 kb) [file 11556_2019_216_MOESM1_ESM.tif]
